# The clinical value of serum sST2 and cfDNA in guiding evidence-based nursing care for children with severe pneumonia complicated by myocardial damage

**DOI:** 10.5937/jomb0-52085

**Published:** 2025-07-04

**Authors:** Meng Du, Haoran Jia, Tingting Zhao, Ye Liu, Dexing Wang, Weiwei Wang

**Affiliations:** 1 Capital Medical University, Baoding Hospital of Beijing Children's Hospital, Neonatology, Baoding, China; 2 Capital Medical University, Baoding Hospital of Beijing Children's Hospital, Pediatrics, China

**Keywords:** sST2, cfDNA, severe pneumonia, myocardial damage, evidence-based nursing, sST2, cfDNA, teška pneumonija, oštećenje miokarda, nega zasnovana na dokazima

## Abstract

**Background:**

To investigate how serum sST2 and cfDNA can be used to inform evidence-based nursing practices for children with severe pneumonia and myocardial damage.

**Methods:**

100 children with severe pneumonia complicated with myocardial damage were recruited as research subjects. After assessing serum sST2 and cfDNA concentrations, the individuals were categorised into a control cohort (receiving standard treatment, n=50) and an experimental cohort (receiving evidence-based treatment guided by serum sST2 and cfDNA markers, n=50). Collected were the general details of the two patient groups. Biochemical analysis of patient serum sST2 and cfDNA changes was performed before and after care. Echocardiography was used to measure the left ventricular ejection fraction (LVEF) and left ventricular internal diameter (LVIDd) both before and after treatment of the patient. Levels of procalcitonin and C-reactive protein were assessed using enzyme-linked immunosorbent assay (ELISA) before and after patient treatment, while the white blood cell count in blood samples was determined using an automated haematology analyser. The patients' pneumonia resolution and length of hospital stay were compared. Patient satisfaction with care plans was compared through rating questionnaires.

**Results:**

The general information of the two groups of patients showed no significant difference (P>0.05). Before receiving nursing care, there were no significant variations in serum sST2 and cfDNA levels among the two patient groups (P>0.05). Following the nursing period, the observation group exhibited decreased serum sST2 and cfDNA levels compared to the control group (P<0.05). Before nursing care, there were no significant variations in left ventricular ejection fraction and left ventricular internal diameter in diastole among the two cohorts of patients (P>0.05). Following nursing, the observation group exhibited a higher LVEF than the control group and a smaller LVIDd (P<0.05). Before receiving nursing care, there were no significant variations in procalcitonin, hs-CRP, and white blood cell count between the two patient groups (P>0.05). Following care, the observation group exhibited decreased levels of procalcitonin, hs-CRP, and white blood cell count compared to the control group (P<0.05). The pneumonia remission and hospitalisation duration in the observation group were significantly shorter than in the control group (P<0.05).

**Conclusions:**

The importance of serum sST2 and cfDNA indicators in evidence-based nursing for children with severe pneumonia and myocardial damage is highlighted, showing significant improvement in treatment outcomes and patient satisfaction, confirming the crucial role of these biomarkers in enhancing nursing care plans.

## Abstract (sr)

**Uvod:**

Cilj je bio da se istraži kako se serumski sST2 i cfDNA mogu koristiti za unapređenje nege zasnovane na dokazima kod dece sa teškom pneumonijom i oštećenjem miokarda.

**Metode:**

U istraživanje je uključeno 100 dece sa teškom pneumonijom komplikovanom oštećenjem miokarda. Nakon procene koncentracija serumskog sST2 i cfDNA, ispitanici su podeljeni u kontrolnu grupu (koja je primala standardni tretman, n=50) i eksperimentalnu grupu (koja je primala tretman zasnovan na dokazima, vo|en markerima serumskog sST2 i cfDNA, n=50). Prikupljeni su opšti podaci o obe grupe pacijenata. Biohemijska analiza promena nivoa serumskog sST2 i cfDNA sprovedena je pre i nakon nege. Ehografijom su mereni frakcija izbacivanja leve komore (LVEF) i unutra{nji dijametar leve komore u dijastoli (LVIDd) pre i nakon tretmana. Nivoi prokalcitonina i C-reaktivnog proteina (hs-CRP) određivani su metodom ELISA pre i nakon tretmana, dok je broj leukocita u uzorcima krvi odre|en pomoću automatskog hematološkog analizatora. Upoređeni su stepen remisije pneumonije i dužina hospitalizacije, a zadovoljstvo pacijenata planom nege procenjeno je putem upitnika.

**Rezultati:**

Opšti podaci obe grupe pacijenata nisu pokazali značajnu razliku (P>0,05). Pre sprovođenja nege, nivo serumskog sST2 i cfDNA se nije značajno razlikovao između grupa (P>0,05). Nakon perioda nege, eksperimentalna grupa je imala niže nivoe serumskog sST2 i cfDNA u poređenju sa kontrolnom grupom (P<0,05). Pre nege, LVEF i LVIDd se nisu značajno razlikovali između dve grupe pacijenata (P>0,05). Nakon nege, eksperimentalna grupa imala je vi{i LVEF i manji LVIDd u pore|enju sa kontrolnom grupom (P<0,05). Pre sprovođenja nege, nije bilo značajnih razlika u nivoima prokalcitonina, hs-CRP i broju leukocita izme|u grupa (P>0,05). Nakon nege, eksperimentalna grupa imala je niže nivoe prokalcitonina, hs-CRP i broj leukocita u poređenju sa kontrolnom grupom (P<0,05). Period remisije pneumonije i dužina hospitalizacije su bili značajno kraći u eksperimentalnoj grupi u poređenju sa kontrolnom grupom (P<0,05).

**Zaključak:**

Serumski sST2 i cfDNA pokazali su se kao ključni indikatori u vođenju nege zasnovane na dokazima kod dece sa teškom pneumonijom i oštećenjem miokarda. Njihova upotreba doprinosi značajnom poboljšanju ishoda lečenja i zadovoljstva pacijenata, potvrđujući njihovu važnu ulogu u unapređenju plana nege.

## Introduction

With the progress of medical science and technology, evidence-based nursing has become an important strategy to improve medical quality and patient safety [1]. Accurate monitoring and personalised management are crucial, especially when treating severe pneumonia with myocardial damage [2]. Severe pneumonia is not only a common disease in children’s intensive care units but also the risk of myocardial damage is significantly increased, which poses a serious threat to children’s life and health [3] [4]. Hence, discovering efficient biomarkers for directing clinical care and nursing is crucial in enhancing children’s treatment outcomes and prognosis. Serum sST2 and cfDNA, as biomarkers widely concerned in the study of cardiology and inflammatory diseases in recent years, have gradually become evident in their roles in myocardial damage and apoptosis [5] [6] [7]. During times of stress, the heart releases SST2, a soluble receptor whose elevated levels are strongly linked to the severity of heart disease and an adverse prognosis. Cell-free DNA is a fragment of DNA released into the bloodstream through cell death, and variations in its levels can indicate the extent of inflammation and tissue injury in the body. Therefore, the joint monitoring of these two markers provides a new perspective and method for evaluating myocardial damage. In recent years, evidence-based nursing strategies based on biomarkers have gradually become the focus of clinical research [8] [9]. By accurately monitoring serum sST2 and cfDNA levels, medical staff can more accurately evaluate the changes in children’s condition and adjust the treatment plan to realise personalised treatment and nursing [10] [11] [12]. The evidence-based nursing approach can enhance patient and family satisfaction, improve treatment outcomes, and elevate the quality of medical care. However, although the clinical application prospect of serum sST2 and cfDNA is broad, the application research in children with severe pneumonia complicated with myocardial damage is relatively limited, especially their specific role and value in evidence-based nursing need to be further explored [13] [14] [15] [16]. This study investigated the soluble tumour suppressor protein 2 (sST2) expression level in specific diseases and its correlation with clinical outcomes. The central hypothesis is that sST2 levels are significantly higher in patients with the studied disease than in healthy controls and that an increase in sST2 is associated with disease severity and poor prognosis. This study aims to enhance the utilisation of evidence-based nursing in treating critically ill children, ultimately advancing clinical medicine and enhancing the quality of medical services. The research innovation is to combine bio markers with clinical nursing and introduce serum sST2 and cfDNA as biomarkers into the nursing of children with severe pneumonia complicated with myocardial injury to realise the formulation and adjustment of a personalised treatment plan. This approach combines real-time monitoring of biomarkers with care practices to improve heart function, reduce inflammation levels and shorten the time to pneumonia remission in children.

## Materials and methods

### General information

A total of 100 children with severe pneumonia with myocardial injury who were admitted to the pediatric Internal Medicine Department of our hospital from January 2022 to October 2023 were recruited as study subjects. Patients ranged in age from 1 to 8 years, including 57 men and 43 women. After assessing serum sST2 and cfDNA levels, patients were divided into a control group (receiving standard care, n=50) and an observation group (receiving evidence-based care based on serum sST2 and cfDNA indices, n=50), with “care practice” referring to the specific behaviours and approaches that care, professionals, use to provide care to patients in a clinical setting. It includes assessing a patient’s health, developing and implementing a care plan, providing daily care, conducting health education, monitoring changes in the patient’s condition, and working with other healthcare team members. Before participation in the study, all patients or their legal guardians were notified of the program and provided their signature on the informed consent form.

Regarding the association between phosphatase pneumonia and myocardial damage, although evidence of myocardial damage observed in children with pneumococcal pneumonia is rare, some studies have suggested that severe pneumonia may lead to systemic inflammatory responses that can cause myocardial damage. For example, Mei Y et al. [17] proposed a research method targeting the risk factors of myocardial injury in children with severe pneumonia and obtained significant results. The study analysed the medical records of 408 children with severe pneumonia and found that 50 (12.3%) had combined myocardial injury. Analysis showed that young age, concomitant hypoxemia, hypokalemia, hypo proteinemia, respiratory failure, decreased pH, de creased PaO_2_, decreased HCO_3_^-^ concentration, and increased procalcitonin and C-reactive protein were independent risk factors in this patient population. At the same time, complicated myocardial injury has been identified as an independent risk factor affecting the prognosis of children with severe pneumonia [17].

The study was designed as a retrospective analysis to explore the impact of specific variables on clinical outcomes based on previous data. Retrospective analysis searches patients’ historical medical records to assess differences and associations between different groups. The study classification criteria were based on clinical features and treatment protocols. The clinical features include age, gender, and underlying diseases (hypertension, diabetes, etc.). Patients were grouped according to the type of treatment or medication they received, and patients with different treatment plans were divided into different groups. Because the study was retrospective, randomised control was not applicable. In the retrospective analysis, the study tried to control the disturbing variables. It used statistical methods (such as multiple regression analysis) to adjust for possible confounding factors to enhance the reliability and validity of the results.

Admission criteria: Patients with pneumococcal infection were selected as study subjects. Children between the ages of 0 and 15 years, regardless of gender, who meet the diagnostic criteria for severe pneumonia and show signs of myocardial damage based on clinical symptoms and test results are admitted to the hospital. To ensure that the cause of pneumonia is clear, all patients undergo a series of etiological tests on admission, including blood cultures, sputum bacterial cultures, and rapid antigen tests to identify the specific agent of infection. Imaging tests, such as chest X-rays or CT scans, are also used to assess the lungs’ condition and comprehensively assess clinical symptoms. Experienced pediatric respiratory doctors diagnosed all cases to ensure a precise diagnosis of the nature of the pneumonia.

sST2, which is produced by splicing the ST2 gene, lacks a transmembrane region and is, therefore, present in soluble form in plasma and interstitial fluid. The transmembrane subtype of ST2 (ST2L) has a complete transmembrane region and can bind to IL-33 through the cell surface, thereby inhibiting IL-33-mediated Th2-type immune response. sST2 is overexpressed in a variety of cells (such as mast cells, macrophages, fibroblasts and cardiomyocytes) under different conditions (such as infection, inflammation, cardiac stress, tumours, etc.) [18].

Inclusion criteria: meet the diagnostic criteria for community-acquired pneumonia (CAP) or hospital-acquired pneumonia (HAP); Had not received antibiotics within 48 hours before hospitalisation; The severity of clinical symptoms of severe pneumonia was evaluated using the CPIS scoring system, including cough (dry or wet cough), fever (>38 ), shortness of breath (respiratory rate > 30 times/min), chest pain, etc. Increased white blood cell count (>12,000 /μL or <4,000/μL), increased procalcitonin levels, oxygen saturation (<90%), positive sputum culture results; Showing shadow or infiltration of the lungs; Pneumonia-related symptoms developed within 48 hours of hospitalisation; The total CPIS score of more than 6 is considered as severe pneumonia.

Exclusion criteria: The patient had serious complications, such as acute renal failure and severe liver failure; Patients with serious chronic respiratory diseases, chronic heart diseases and other basic diseases; Previous history of heart disease such as myocarditis and myocardial infarction or other serious chronic diseases; Patients whose guardians refused to sign the informed consent were excluded.

### Medical ethics issues

The research received approval from our hospital’s Ethics Committee, which ensured that all procedures adhered to ethical norms and regulations. Participants provided written consent before participating in the research.

### Methods

### Patient treatment plan

Patients were divided into a control group, which received standard care, and an observation group, which received individualised evidence-based care.

Standard of care: Based on the child’s clinical presentation and laboratory findings, targeted antibiotic therapy is administered, with the initial use of broad-spectrum antibiotics and necessary adjustments based on bacterial culture results. Care provides supportive treatment, including fluid resuscitation to maintain water and electrolyte balance and prevent dehydration, with intravenous fluids if necessary. At the same time, children with hypoxemia are given oxygen therapy, using a nasal catheter or mask to provide the appropriate amount of oxygen to ensure that the blood oxygen saturation is maintained within a safe range (usually >92%). Vital signs, including body temperature, heart rate, respiratory rate and blood pressure, are regularly monitored to assess changes in the condition. At the same time, fever and discomfort are controlled by administering antipyretic and analgesic drugs (such as acetaminophen), and expectorants are used when necessary to improve airway patency. The caregiver should observe the patient for symptoms, record any changes, and communicate with the healthcare team to adjust the care plan to ensure that the patient is fully and effectively managed.

Evidence-based care options: In the observational group of evidence-based care protocols, serum sST2 and cfDNA levels were first assessed by the first blood monitoring after admission. ST2 and cfDNA detection can provide important biomarker data to guide interventions for children with severe pneumonia and myocardial injury. Based on the test results, if sST2 levels are significantly elevated, cardioprotective interventions are implemented, fluid management regimens are adjusted to reduce cardiac load, and positive inotropic drugs are considered to improve cardiac function. If cfDNA levels are high, there is an emphasis on strengthening anti-infective treatment, possibly adjusting the type and dosage of antibiotics to deal with potential bacterial infections. During care, cardiac function monitoring was performed regularly, including left ventricular ejection fraction (LVEF) and left ventricular end-diastolic diameter (LVIDd) assessed by echocardiography, and the care plan was dynamically adjusted to respond to changes in the patient’s condition. In addition, patients are given individualised health education, which instructs families on how to observe and respond to potential symptom changes and provides psychological support to improve patient and family satisfaction and compliance. The care team should also hold regular case discussion sessions to share patient data and biomarker results, ensuring that all care measures are based on the latest clinical evidence to maximise the patient’s overall health outcomes and recovery speed.

### Detection of serum sST2 and cfDNA levels

Before the nursing (the first day after admission) and after the nursing (about 20 days later), 5ml of whole blood was collected from the patient’s vein, and the serum was separated by centrifugation at 3000rpm for 10 minutes. Following that, the concentration of sST2 was assessed using an ELISA kit, and cfDNA was isolated using a specialised kit and subsequently analysed quantitatively using qPCR.

sST2 is a biomarker of cardiac stress response and is associated with the prognosis of myocardial injury, heart failure, and heart disease. Elevated concentrations usually indicate a state of stress in the heart, especially an increase in cardiomyocyte damage or heart failure. High levels of sST2 are associated with a poorer prognosis and can help assess the severity of myocardial damage and prognostic risk in patients. cfDNA is a piece of DNA released into the blood after tissue damage. In the event of myocardial injury, cardiomyocyte death results in the release of cfDNA, reflecting the extent of damage to the heart. Levels of cfDNA could be used for early detection of heart muscle damage and to monitor a patient’s heart health during treatment.

sST2, which is produced by splicing the ST2 gene, lacks a transmembrane region and is, therefore, present in soluble form in plasma and interstitial fluid. The transmembrane subtype of ST2 (ST2L) has a complete transmembrane region and can bind to IL-33 through the cell surface, thereby inhibiting IL-33-mediated Th2 type immune response. sST2 is overexpressed in various cells (such as mast cells, macrophages, fibroblasts and cardiomyocytes) under different conditions (such as infection, inflammation, cardiac stress, tumours, etc.).

### Echocardiographic detection

Patients need to be evaluated for myocardial damage before and after receiving evidence-based care. The definition of myocardial injury usually refers to the damage or death of heart muscle cells, resulting in cardiac dysfunction [19]. This damage can be confirmed in several ways, including elevated myocardial markers, imaging tests (such as echocardiography), and the patient’s clinical symptoms (such as chest pain, dyspnea, and weakness). There is a complex association between myocardial damage and severe pneumonia. Severe pneumonia can lead to a systemic inflammatory response, and the release of cytokines may cause direct or indirect damage to cardiomyocytes. Severe pneumonia is often accompanied by a state of hypoxia, which may cause myocardial metabolic disorders, resulting in myocardial cell damage.

Specific indicators of myocardial injury are mainly evaluated by echocardiography, including left ventricular ejection fraction (LVEF) and left ventricular end-diastolic diameter (LVIDd). LVEF is a key index to assess the pumping function of the heart and reflects the contractile function of the myocardium. LVIDd reflects the diastolic chamber size of the heart and can reflect the status of myocardial diastolic function. By monitoring these two indicators, the study could quantitatively assess the severity of myocardial damage.

An echocardiogram was performed on the child before and after care, during which the child remained flat to ensure the stability and accuracy of the images. An experienced ultrasound technician uses standard two-dimensional echocardiography equipment to collect heart images. According to the AHA’s measurement guidelines, the LVEF was calculated using Simpson’s double section method using device software, while LVIDd was measured directly from a two-dimensional image.

### ELISA and white blood cell count

Blood samples of children were obtained by venous blood sampling and divided into two parts. Part of it was used for ELISA detection. The special procalcitonin and hs-CRP ELISA kit were used to operate according to the steps provided by the manufacturer. Finally, the concentrations of procalcitonin and hs-CRP in the sample were calculated. The other part of the blood sample was used for an automatic blood analyser to count white blood cells, and the instrument automatically processed the sample and provided the total number of white blood cells.

### Clinical symptom relief and hospitalisation time

The duration of remission and hospital stay for pneumonia patients was recorded. Definition of remission time of pneumonia: from the time when children began to receive evidence-based nursing to the time when clinical symptoms of pneumonia (such as fever, cough, dyspnea, etc.) completely disappeared, and chest imaging examination showed that inflammation was absorbed, it was calculated as remission time of pneumonia. Measurement of hospitalisation time: record the total days from admission to discharge of children as a direct indicator of hospitalisation time. The data on remission time and hospitalisation time of pneumonia were collected through the medical records and follow-up of children.

### Satisfaction score

A scoring questionnaire was designed to comprehensively assess the satisfaction of children with severe pneumonia and myocardial damage before and after receiving evidence-based nursing care. The survey included three main aspects: treatment impact, quality of nursing care, and hospital stay experience, with each category scored out of 100 to gauge overall satisfaction among family members. After the end of evidence-based nursing, questionnaires were distributed to the families of children, and data were collected by face-to-face or electronic means, ensuring the universality and accuracy of the evaluation.

### Statistical analysis

Graph Pad Prism software (San Diego, USA) was used for statistical analysis. The Shapiro-Wilk test assessed the normal distribution of data values within each group. Parameter data underwent one-way ANOVA followed by the Tukey test, while nonparametric data underwent analysis using the Kruskal-Wallis test followed by the Dunn multiple comparison test. For all analyses, p 0.05 was considered significant.

## Results

### Statistics of general data of patients

According to the general data of patients, the ratio of males to females in the control group was 28:22, the average age was 4.24±1.33 years, the average BMI was 15.36±1.24 kg/m^2^, and the duration of symptoms before admission was 4.27±0.48 days. The ratio of males to females in the observation group was 27:23, with an average age of 4.40±1.17 years and an average age of 14.98±1.56 kg/m^2^. The duration of symptoms before admission was 4.51±0.36 days. There was no difference in general data between the two groups (P>0.05) (Table 1).

**Table 1:** Statistics of general data of patients (x̄±s).

Project	Control group<br>(n=50)	Observation group<br>(n=50)	T value /χ^2^ value	P value
Gender (male: female)	28:22	27:23	0.000	1.000
Age (years)	4.24±1.33	4.40±1.17	0.6387	0.5245
BMI (kg/m^2^)	15.36±1.24	14.98±1.56	1.3484	0.1808
Duration of symptoms before<br>admission (days)	4.27±0.48	4.31±0.36	0.4714	0.6385
Serum sST2 (ng/mL)	36.57±6.33	35.45±5.24	3.208	0.506
cfDNA (ng/mL)	122.67±15.49	119.54±17.38	1.005	0.387
LVEF (%)	41.00±0.80	41.30±0.90	1.761	0.813
LVIDd (mm)	41.46±3.45	42.30±3.27	3.051	0.562
Procalcitonin (ng/mL)	0.47±0.12	0.48±0.14	2.661	0.335
hs-CRP (mg/L)	25.48±3.78	25.67±3.66	0.725	0.725
White Blood Cell Count (×10^9^/L)	12.57±2.14	12.49±2.05	3.793	0.585

### sST2 and cfDNA level analysis

Biochemical analysis revealed no significant difference in serum sST2 and cfDNA levels between the two groups Baseline data (P>0.05), but post-nursing, the observation group showed lower levels of serum sST2 and cfDNA compared to the control group (P<0.05) (Table 2).

**Table 2:** Analysis of sst2 and cfDNA levels.

Groups	sST2 (ng/mL)		cfDNA (ng/mL)	
	Baseline data	After nursing	Baseline data	After nursing
Control group (n=50)	36.57±6.33	34.31±5.44	122.67±15.49	118.53±13.66
Observation group (n=50)	35.45±5.24	28.26±3.20	119.54±17.38	92.37±10.55
* T value *	3.208	11.179	1.005	9.274
* P value *	0.506	0.001	0.387	0.005

### Echocardiographic detection

Based on the baseline data intervention, there was no difference between the two groups’ abnormal rate of electrocardiogram and troponin (P>0.05). After the nursing intervention, the abnormal rate of electrocardiogram and troponin in the observation group were lower than those in the control group (P<0.05) (Table 3). Based on the baseline data, there was no difference between LVEF and LVIDd in the two groups (P>0.05). After nursing, LVEF in the observation group was higher than that in the control group, and LVIDd was smaller (P<0.05) (Table 4).

**Table 3:** Comparison of electrocardiogram abnormality rate and troponin in two groups.

Groups	Abnormal electrocardiogram rate (%)		Troponin (ng/mL)	
	Baseline data	After nursing	Baseline data	After nursing
Control group (n=50)	20	14	0.095±0.023	0.090±0.020
Observation group (n=50)	23	2	0.093±0.015	0.050±0.012
* T/χ^2^ value *	0.163	9.003	0.515	12.127
* P value *	0.686	0.001	0.562	0.001

**Table 4:** Comparison of LVEF and LVIDd in two groups.

Groups	LVEF (%)		LVIDd (mm)	
	Baseline data	After nursing	Baseline data	After nursing
Control group (n=50)	41.0±0.8	42.8±1.2	41.46±3.45	40.62±3.22
Observation group	41.3±0.9	48.2±1.8	42.30±3.27	35.51±2.15
* T value *	1.761	6.099	3.051	13.064
* P value *	0.813	<0.001	0.562	<0.001

### Characteristics of severe pneumonia

In the characteristic detection of severe pneumonia, the CT results (normal) and Positive rate of bacterial culture of the two groups had no significant difference between the two groups Baseline data (P>0.05). There was a significant difference in the nursing group (P<0.05). The X-ray result and Operation rate (%) of the Control group were not significantly different before and after care (P>0.05). The X-ray result and Operation rate (%) of the Observation group were not significantly different in the pre-nursing group (P>0.05). There was a significant difference in the group after nursing (P<0.05) (Table 5).

**Table 5:** Comparison of characteristics of severe pneumonia.

Index		Control group (n=50)	Observation group	* P *
CT results<br>(normal)	Baseline data	20 (40%)	22 (44%)	0.839
	After nursing	35 (70%)	45 (90%)	0.024
* P *		0.005	<0.001	/
X-ray result	Baseline data	18 (36%)	20 (40%)	0.837
	After nursing	32 (64%)	42 (84%)	0.040
* P *		0.093	<0.001	/
Positive rate of<br>bacterial culture	Baseline data	28 (56%)	26 (52%)	0.841
	After nursing	10 (20%)	5 (10%)	0.263
* P *		<0.001	<0.001	/
Operation rate (%)	Baseline data	5 (10%)	6 (12%)	1.000
	After nursing	3 (6%)	0 (2%)	0.241
* P *		0.712	0.035	/

### Inflammation level analysis

Based on the baseline data, there was no difference in procalcitonin, hs-CRP, or white blood cell count between the two groups (P>0.05). After nursing, the levels of procalcitonin, hs-CRP and white blood cell count in the observation group were lower than those in the control group (P<0.05) (Table 6).

**Table 6:** Analysis of inflammation level.

Groups	Procalcitonin (ng/mL)		hs-CRP (mg/mL)		Leukocyte (×10^9^/L)	
	Baseline data	After nursing	Baseline data	After nursing	Baseline data	After nursing
Control group (n=50)	0.47±0.12	0.46±0.13	25.48±3.78	25.14±3.25	12.57±2.14	12.42±2.11
Observation group (n=50)	0.48±0.14	0.29±0.08	25.67±3.66	19.45±2.41	12.49±2.05	9.37±1.13
* T value *	2.661	13.288	3.024	11.305	3.793	9.644
* P value *	0.335	0.002	0.725	0.001	0.585	0.005

### Comparison of remission and hospitalisation time of patients with pneumonia

**Figure 1:**
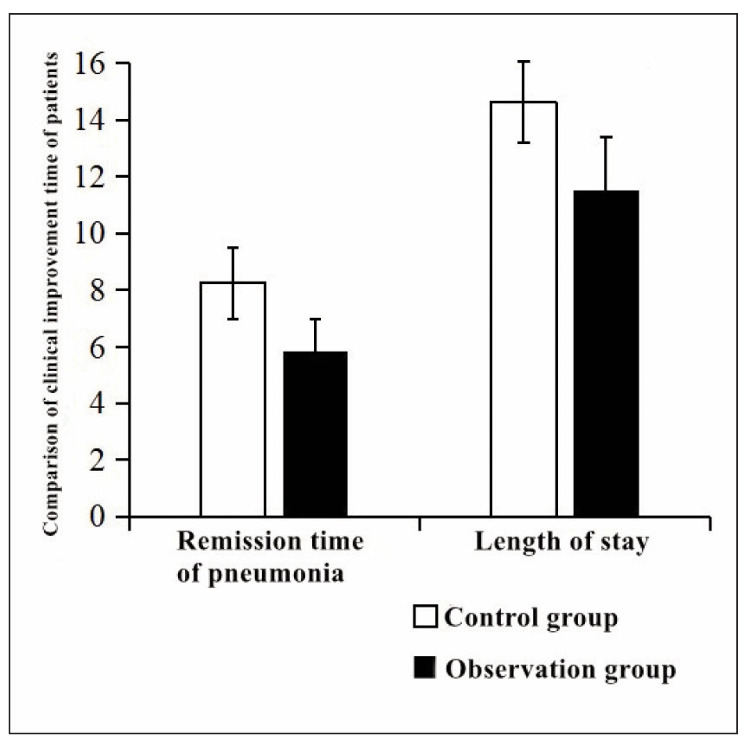
Remission time and hospitalisation time of patients with pneumonia.

Comparing the remission time and hospitalisation time of patients with pneumonia, the remission time and hospitalisation time in the observation group were shorter than those in the control group (P<0.05) (Figure 1).

### Satisfaction survey and analysis

**Figure 2:**
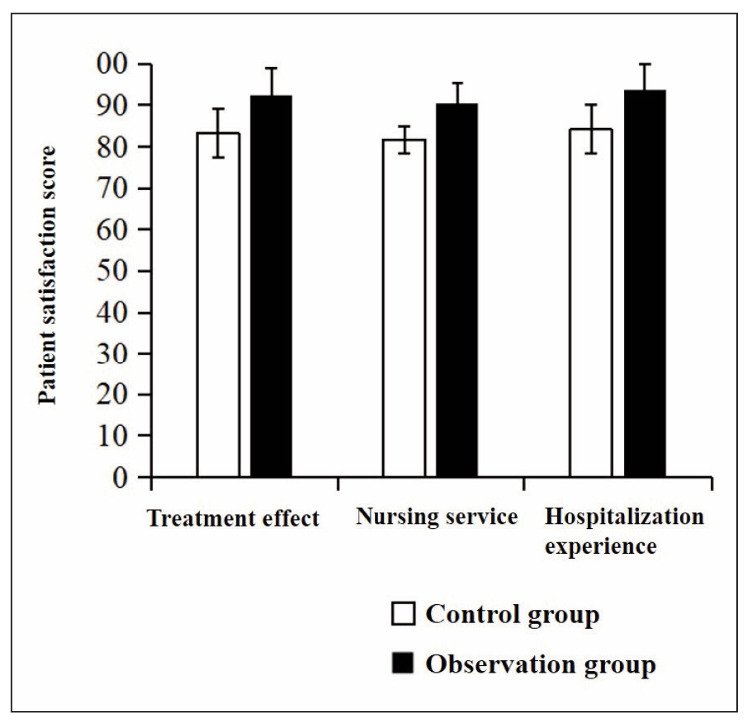
Patient satisfaction score.

The scores of treatment effect satisfaction, nursing service satisfaction and hospitalisation experience satisfaction in the observation group were higher than those in the control group (P<0.05) (Figure 2).

## Discussion

Evidence-based nursing involves using the most reliable evidence to guide clinical decision-making to deliver individualised and optimal nursing care [20]. In this study, two biomarkers, serum sST2 and cfDNA, were incorporated into the nursing decision-making process, and an evidence-based nursing strategy was formed for children with severe pneumonia complicated with myocardial damage. The execution of this plan has greatly enhanced the heart function of children, as shown by the rise in LVEF and the reduction in LVIDd, demonstrating that evidence-based nursing tactics have successfully boosted the restoration of cardiac function. Evidence-based nursing interventions can decrease inflammation in pediatric patients and enhance overall health by lowering procalcitonin, hs-CRP, and white blood cell count. The reduction in these biomarkers shows an improvement in inflammation. It demonstrates the patient’s favourable reaction to treatment, confirming the crucial role of evidence-based nursing in decreasing inflammation and enhancing recovery. The optimisation of evidence-based nursing strategies based on biomarkers not only improves the scientificity and effectiveness of nursing but also provides patients with more accurate and personalised treatment programs. Through continuous monitoring and analysis of biomarkers, medical staff can adjust nursing measures in real-time, maximise the recovery of patient’s heart function, reduce the inflammatory reaction, and thus improve patients’ prognosis and quality of life. This strategy introduces a novel approach to caring for children with severe pneumonia and myocardial damage, highlighting the significance of evidence-based nursing in contemporary healthcare [21].

The study found that using serum sST2 and cfDNA indexes to guide nursing strategies significantly enhanced the clinical outcomes for children with severe pneumonia and myocardial damage and increased patient and family satisfaction. Enhancing patient satisfaction with treatment outcomes, nursing services, and hospital experiences underscores the crucial role of evidence-based nursing in delivering patient-centred care while emphasising the significance of tailored and accurate medical treatment. By introducing biomarkers such as serum sST2 and cfDNA, an evidence-based nursing strategy can provide patients with more precise treatment monitoring and personalised nursing plans so that patients and families can feel more attention and respect during treatment, thus improving their overall evaluation and satisfaction with medical services. This study has a far-reaching impact on clinical practice. Demonstrating the practicality of serum sST2 and cfDNA in managing children with severe pneumonia and myocardial damage can enhance the use of biomarkers in clinical decision-making, offering clinicians a more precise and evidence-based treatment approach. The successful implementation of evidence-based nursing strategy provides empirical support for clinical nursing, which proves the effectiveness of individualised nursing planning based on the best evidence and helps to popularise the application of evidence-based nursing in other disease management [22]. It was established evidence-based nursing indicators for airway management in adult critically ill patients, which provided a useful reference for evidence-based nursing strategies in our study. This indicates that the application of biomarkers to guide nursing practice in children’s critical care also has the potential to be popularised [23]. In the future, we should continue to explore how to combine these biomarkers with other clinical indicators to form a comprehensive nursing evaluation system to improve nursing quality and patient prognosis [24]. Lu et al. [25] explored the correlation between blood parameters and clinical features in patients with pneumonia, highlighting the potential role of blood indicators in monitoring disease progression and revealing the interaction between different infectious diseases. This is consistent with the research, which shows that serum sST2 and cfDNA, as biomarkers, can reflect the degree of myocardial injury and be closely related to the clinical manifestations related to pneumonia, providing a more accurate basis for nursing intervention [24].

In modern medicine, the role of biomarkers is increasingly prominent, especially in monitoring the progress of diseases and evaluating the therapeutic effect. The study found that children with severe pneumonia and myocardial damage had significantly lower serum sST2 and cfDNA levels when evidence-based nursing measures were implemented, confirming the importance of these biomarkers in monitoring myocardial damage. SST2 is a receptor whose level rises when the heart is stressed or injured, which can reflect the heart’s stress state and injury degree. It is commonly utilised in the assessment and prediction of heart conditions like heart failure and acute myocardial infarction as a reliable indicator of cardiac stress and damage. In this study, the decrease in sST2 level suggests that the degree of heart injury is reduced, which may be due to evidence-based nursing measures to improve heart function and reduce heart stress effectively. As a direct sign of cell death and tissue damage, the change of cfDNA concentration directly reflects the degree of cell apoptosis and tissue damage in vivo. In this study, the decrease in cfDNA level shows that evidence-based nursing measures may effectively reduce tissue damage and help to repair damaged tissue quickly, thus reducing cell apoptosis and injury. Viglianisi G et al. [26] focused on cfDNA as a biomarker for evaluating the development of chronic diseases, emphasising that dynamic changes in cfDNA are crucial in monitoring the disease process and are consistent with changes in cfDNA levels in the study, which further demonstrates the importance of cfDNA in acute diseases, especially in the course of infection. Therefore, the integration of these research results indicates that cfDNA not only has potential value in monitoring chronic diseases but cannot be ignored in the application of acute diseases [25]. Monitoring these biomarkers not only provides immediate feedback on the degree of myocardial damage and therapeutic effect in clinic, but also provides important reference information for doctors to help them adjust the treatment plan to achieve better therapeutic effect. In addition, monitoring sST2 and cfDNA may also help predict the prognosis of the disease and provide more accurate treatment and care for patients [23] [24] [25] [26] [27] [28]. Therefore, these biomarkers play a vital role in the monitoring and treatment evaluation of myocardial damage and provide an effective tool for clinicians to optimise patient management and improve the treatment effect.

## Conclusion

In summary, this research emphasises the importance of evidence-based nursing utilising serum sST2 and cfDNA markers in managing severe pneumonia with myocardial damage in children. By monitoring the changes of these two biomarkers in real-time with the proposed method, medical personnel can more accurately assess the severity of myocardial injury and inflammatory response to adjust the treatment plan and implement personalised care measures. This cannot only effectively improve heart function and reduce the level of inflammation in children but also significantly shorten the length of hospital stay and improve patient satisfaction, providing a scientific basis for nursing practice based on biomarkers and promoting the development of personalised medicine. Therefore, this study provides clinicians with a new tool to improve the overall treatment outcome and prognosis of children with severe pneumonia complicated with myocardial injury and has broad clinical application potential. Still, the study has some limitations. The shortcomings of the study are mainly reflected in the small sample size, which is limited to the rarity of children with severe pneumonia complicated with myocardial injury, which may affect the universality and generalisation of the results. In addition, the failure to analyse serum sST2 and cfDNA use in other infectious diseases limits their applicability in a broader range of clinical Settings. Therefore, future studies should consider expanding the sample size to enhance statistical power and conduct multicenter, multi-book prospective studies to validate the clinical value of these biomarkers in other types of infections and children of different ages. At the same time, an in-depth exploration of the potential role of sST2 and cfDNA in the surveillance and management of infectious diseases can provide helpful implications and guidance for broader evidence-based care practice.

## Dodatak

### Ethics approval and consent to participate

The study was approved by the local ethics committee of the Beijing Children’s Hospital, Capital Medical University. All experiments followed relevant guidelines and regulations, such as the Declaration of Helsinki.

### Consent for publishing

Informed consent was obtained from all subjects and/or their legal guardian(s).

### Availability of data and materials

The original contributions presented in the study are included in the article.

### Funding

Beijing Children’s Hospital Affiliated to Capital Medical University, Baoding Hospital. The value of serum sST2 and cfDNA in predicting myocardial damage in children with severe pneumonia, (No. 2341ZF390).

### Authors’ contributions

MD, HJ, TZ, YL, DW, and WW participated in interpreting results and data collection, drafting the manuscript, studying concept and design, and supervising the study. All authors read and approved the final manuscript.

### Acknowledgements

Not applicable.

### Conflict of interest statement

All the authors declare that they have no conflict of interest in this work.
